# Editorial: Mechanisms of drug resistance in cancer: overcoming barriers with novel pharmacological strategies

**DOI:** 10.3389/fphar.2026.1996202

**Published:** 2026-10-21

**Authors:** Pulak Kar, Donato Colangelo, Maushmi Shailesh Kumar, Maya Guncheva

**Affiliations:** 1 Department of Biological Sciences, SRM University-AP, Amaravati, India; 2 Dipartimento di Scienze della Salute, Università del Piemonte Orientale, Novara, Italy; 3 Somaiya Vidyavihar University, Mumbai, India; 4 Institute of Organic Chemistry with Centre of Phytochemistry, Bulgarian Academy of Sciences Sofia, Sofia, Bulgaria

**Keywords:** cancer drug resistance, pharmacological strategies, precision oncology, targeted therapy, therapeutic resistance

Advances in molecular oncology, targeted therapies, immunotherapy, and precision medicine have transformed cancer treatment over the past 2 decades (Soragni et al., 2025; Hanahan, 2022). However, drug resistance remains a major cause of therapeutic failure, disease recurrence, and cancer-related mortality. Tumour cells possess a remarkable ability to adapt to therapeutic pressure through diverse mechanisms, including genetic mutations, epigenetic remodelling, metabolic rewiring, altered drug uptake, ATP-binding cassette (ABC) transporter-mediated drug efflux, dysregulated DNA damage responses, activation of compensatory signalling pathways, and interactions with the tumour microenvironment, all of which can contribute to reduced drug efficacy and therapeutic failure (Holohan et al., 2013; Ge et al., 2025; Sun et al., 2026). Consequently, understanding the molecular basis of therapeutic resistance and identifying strategies capable of restoring treatment sensitivity have become central objectives of modern cancer research (Hanahan, 2022; Holohan et al., 2013; Vasan et al., 2019).

The Research Topic *“Mechanisms of Drug Resistance in Cancer: Overcoming Barriers with Novel Pharmacological Strategies”* brings together studies exploring resistance mechanisms and emerging therapeutic strategies. Collectively, these contributions highlight the complexity of resistance across diverse malignancies and identify novel pharmacological approaches to improve clinical outcomes (Figure 1).

**FIGURE 1:**
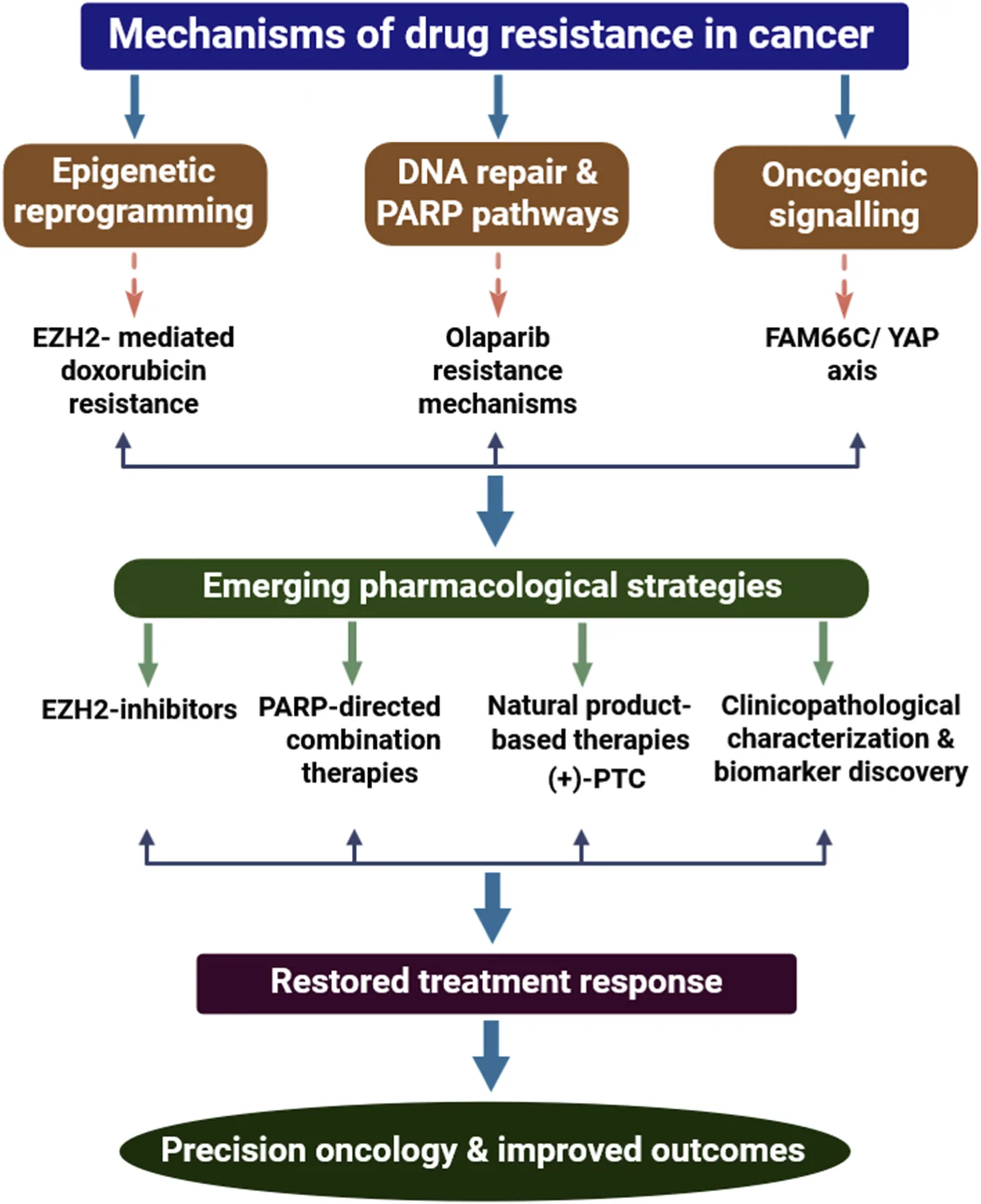
Mechanisms of drug resistance and emerging pharmacological strategies highlighted in this Research Topic. Cancer cells develop therapeutic resistance through diverse and interconnected mechanisms, including epigenetic reprogramming, dysregulation of DNA damage response pathways, activation of oncogenic signalling networks, and tumour heterogeneity. The studies included in this Research Topic highlight representative resistance mechanisms, including EZH2-mediated doxorubicin resistance, Olaparib resistance associated with DNA repair pathways, and FAM66C/YAP-driven chemoresistance, as well as the importance of clinicopathological characterization and biomarker discovery. Emerging pharmacological approaches, including EZH2 inhibition, PARP-directed combination therapies, and natural product-based therapies such as (+)-PTC, may restore therapeutic sensitivity and ultimately contribute to precision oncology and improved patient outcomes.

A recurring theme throughout this Research Topic is the dynamic interplay between cellular adaptation and therapeutic response. Increasing evidence suggests that resistance is not merely a consequence of genetic evolution but also involves profound epigenetic and metabolic reprogramming that enables tumour cells to survive treatment-induced stress (Marine et al., 2020). Recent studies have further identified drug-tolerant persister (DTP) states as a transient and reversible adaptive phenotype that enables tumour cells to survive therapeutic pressure without permanent genetic alterations. Importantly, these non-genetic tolerance states may serve as an intermediate step that facilitates the eventual emergence of stable acquired resistance (Marine et al., 2020; Russo et al., 2024). This concept is exemplified by the article *“EZH2 blockade reverses doxorubicin resistance by inducing metabolic vulnerability and enhancing DNA damage in breast cancer”* (Wang et al.), which demonstrates that pharmacological inhibition of EZH2 enhances DNA damage and promotes metabolic vulnerability in resistant cancer cells, thereby restoring therapeutic sensitivity. These findings further support the importance of epigenetic regulators in therapeutic adaptation and resistance biology. Although recent clinical and regulatory challenges associated with the EZH2 inhibitor tazemetostat highlight the complexities of translating epigenetic therapies into durable clinical benefit, EZH2 remains an important biological regulator and potential therapeutic target warranting continued investigation. Another important theme in this Research Topic is resistance to DNA damage response targeted therapies. Despite the clinical success of poly (ADP-ribose) polymerase (PARP) inhibitors, acquired resistance often limits their long-term benefit (Lord and Ashworth, 2017). Addressing this issue, the article *“Eprenetapopt in Combination with Carboplatin in High-Grade Ovarian and Triple Negative Breast Cancer Cell Lines with Acquired Resistance to Olaparib”* (Pineda et al.) explored the therapeutic potential of eprenetapopt, a small molecule capable of restoring wild-type-like p53 function in TP53-mutant cells and promoting ferroptosis-associated cellular responses, in combination with carboplatin in preclinical Olaparib-resistant models. The reported synergistic activity suggests that rational combination strategies may exploit persistent vulnerabilities in DNA damage response pathways and warrants further investigation as a potential approach to mitigate resistance to targeted therapies (Wang et al., 2026; Wahida and Conrad, 2025). Such approaches emphasize the importance of integrating mechanistic understanding with therapeutic design to achieve more durable clinical responses. The importance of rationally designed therapeutic combinations is further highlighted by the article *“Personalized Boolean Network Dynamic Profiling and Machine Learning Approaches to Identify Combinatorial Treatments for Prostate Cancer”* (Kim et al.), which demonstrates how systems biology and machine-learning approaches can be leveraged to identify patient-specific therapeutic vulnerabilities and optimize combinatorial treatment strategies. These computational frameworks complement experimental approaches and may enable more precise treatment strategies for resistant malignancies. The collection also highlights the role of non-coding RNAs and oncogenic signalling networks in therapeutic responsiveness. In this regard, the article *“LncRNA FAM66C Enhances Gastric Cancer Proliferation and Chemoresistance Through Phosphorylating Nuclear YAP Protein”* (Yang et al.) identifies a novel mechanism through which FAM66C promotes proliferation and chemoresistance by regulating nuclear YAP signalling. These findings expand our understanding of the molecular circuitry governing resistance and emphasise the potential of lncRNA-associated pathways as biomarkers and druggable targets in refractory disease. Resistance to targeted therapies also remains a major challenge in breast cancer management. The review article *“ADC-after-ADC Paradigm: Navigating Cross-Resistance and Sequencing in Breast Cancer Therapy”* (Asare et al.) examines the emerging complexities associated with sequential antibody-drug conjugate (ADC) therapies and highlights the importance of understanding cross-resistance mechanisms, therapeutic sequencing, and patient selection. As ADC-based treatments become increasingly integrated into clinical practice, such insights will be critical for maximizing therapeutic benefit and delaying resistance. Complementing these mechanistic insights, the Research Topic also includes investigations into novel anti-cancer compounds with potential applications in resistant tumours. The article *“The Pterocarpan (+)-PTC Modulates Cytoskeletal Proteins and Induces Apoptosis in Metastatic Castration-Resistant Prostate Cancer: A Proteomic Perspective”* (Neto et al.) demonstrated that (+)-PTC influenced proteins associated with cytoskeletal organization and apoptosis-related signalling, suggesting potential mechanisms underlying its anti-tumour activity in metastatic castration-resistant prostate cancer. Given the clinical burden of advanced prostate cancer, such studies support the exploration of alternative therapeutic strategies, including bioactive natural products. More broadly, overcoming resistance requires understanding both therapeutic vulnerabilities and tumour heterogeneity. The article *“Clinicopathological and Immunohistochemical Analysis of Poorly Differentiated Squamous Cell Carcinoma and Its Impact on Quality of Life”* (Radhakrishnan et al.) demonstrates the value of detailed molecular and pathological assessment in informing treatment decisions and identifying prognostic biomarkers associated with disease progression and supporting improved patient stratification. Expanding the focus beyond tumour cell-autonomous mechanisms, the tumour microenvironment is increasingly recognized as a critical determinant of therapeutic response. The review article *“Beyond Angiogenesis: The Paradoxical Emerging Pro-Tumorigenic Role of Thrombospondin-2”* (Abhay Shah et al.) reveals the complex and context-dependent functions of stromal signalling networks in cancer progression. By emphasizing the multifaceted roles of thrombospondin-2 in tumour biology, this contribution broadens our understanding of resistance-associated microenvironmental factors and identifies new opportunities for pharmacological intervention.

The contributions assembled in this Research Topic reinforce key principles shaping contemporary cancer research. Therapeutic resistance is a dynamic and multifactorial process involving genetic, epigenetic, metabolic, and microenvironmental mechanisms (Soragni et al., 2025). Overcoming resistance will require mechanistically informed combination therapies, longitudinal tumour characterization, and integration of emerging technologies, including multi-omics and artificial intelligence, to identify actionable vulnerabilities. Ultimately, successful clinical translation will depend on continued advances in biomarker discovery, mechanistic understanding, and innovative drug development.

As oncology advances toward increasingly personalized treatment strategies, overcoming drug resistance will remain a major challenge and determinant of long-term therapeutic success. The eight contributions featured in this Research Topic provide complementary perspectives on resistance mechanisms and emerging pharmacological approaches across diverse cancer types. Collectively, they encourage a shift from viewing resistance as an inevitable consequence of therapy to recognizing it as an adaptive process that may reveal new therapeutic opportunities and guide the development of more effective cancer treatments.
